# Iconic Words Are Associated With Iconic Gestures

**DOI:** 10.1111/cogs.70098

**Published:** 2025-08-05

**Authors:** Ell Wilding, Bodo Winter, Jeannette Littlemore, Marcus Perlman

**Affiliations:** ^1^ Department of Linguistics and Communication University of Birmingham

**Keywords:** Iconicity, Gesture, Cognition, Multimodal communication, Sensorimotor semantics, Sensory linguistics, Quantitative methods, Big data

## Abstract

Iconicity ratings studies have established that there are many English words which native speakers judge as “iconic,” that is, as sounding like what they mean. Here, we explore whether these iconic English words are more likely to be accompanied by iconic gestures. We report a large‐scale quantitative study comparing the gesture rate of words rated as high in iconicity (e.g., *swoosh*, *puffy*, *crispy*) to those rated as low in iconicity (e.g., *ordain*, *rejoin*, *grateful*), balancing for perceptual strength, part‐of‐speech, and syllable length. Five thousand seven hundred and twenty‐five tokens from the TV News Archive were coded for whether speakers produced a gesture with the word, and whether the gesture was iconic. The results show that high iconicity words have a higher overall gesture rate (69%) than low iconicity words (56%): specifically, high iconicity words have a higher *iconic* gesture rate (24% vs. 11%). This effect is more pronounced among verbs than adjectives, which we hypothesize may be due to the dynamic nature of verbs. We also find that this result persists when controlling for perceptual and action strength ratings, suggesting that word‐level iconicity is a more important predictor than sensorimotor strength of whether a speaker will use an iconic gesture. We find that some high iconicity words are more likely to occur with iconic gestures when they come with markers of syntactic isolation, suggesting that morphosyntactic behavior is also relevant to iconic gesture production. Our findings demonstrate that iconicity in spoken communication is inherently multimodal, manifesting in both speech and gesture simultaneously, and that iconicity is often psychologically active when speakers use conventionalized iconic words.

## 1 Introduction

The traditional view of spoken language sees words as arbitrary: the forms of words bear no resemblance to their meaning. In contrast, many gestures—most typically, communicative movements of the hands—are iconic, exhibiting resemblance between their form and meaning. Multimodal approaches to language, which understand gesture to be an integral component of spoken communication, have often emphasized a semiotic division of labor (Holler & Levinson, 2019; McNeill, 2005): on this view, words are treated as communicating the propositional content of a message (denotation or description), and co‐speech gestures as communicating the imagistic and analog aspects of a message (depiction). Iconicity, under this framework, is seen as a property that primarily characterizes gesture, rather than speech.

Yet, many spoken languages have extensive vocabularies of iconic words. A prime example is the prevalence across many languages of ideophones, a distinct lexical class of words that vividly depict sensory imagery (Dingemanse, 2012). These words—such as *dzarra‐dzarra* (“scribble, doodle” in Basque) (Ibarretxe‐Antuñano, 2017, p. 202), *ɣììì* (“the sensation of vertigo” in Siwu) (Dingemanse, 2012, p. 661), and *zoɾozoɾot* (“one after another in line” in Japanese) (Dingemanse & Akita, 2017, p. 518)—are often felt to be iconic by native speakers (Kita, 1997; Thompson, Akita, & Do, 2020). Notably, people frequently produce iconic gestures alongside these words, suggesting ideophones and gestures reflect two complementary forms of depiction (Dingemanse, 2013; Kita, 1997).

However, in comparison to most of the world's spoken languages, the words of English and other European languages have been characterized as lacking in iconicity (Perniss & Vigliocco, 2014; Perniss, Thompson, & Vigliocco, 2010), and studies of gesture among speakers of these languages have generally overlooked co‐occurring iconicity in speech. This perception of English as lacking in iconicity has been challenged for some time, with linguists highlighting examples of iconicity in the lexicon and prosody of the English language (Bolinger, 1949; Bolinger & Sears, 1981; Waugh, 1992, 1994). More recently, iconicity ratings and statistical analyses of form‐meaning correlations (Sidhu, Westbury, Hollis, & Pexman, 2021; Winter, Lupyan, Perry, Dingemanse, & Perlman, 2023; Winter & Perlman, 2021b) have built on this research, revealing that English has many more iconic words than traditionally assumed. These findings suggest that the semiotic division of labor between words and gestures, even in English, may not be as distinct as once thought. Like gestures, words may also participate in an imagistic and analog mode of communication alongside their conventional and denotative characteristics.

This view has its challenges, however. Because words rated high in iconicity, such as *swoosh*, *puffy*, *wobbly*, and *gooey*, also have a conventional form‐meaning link, it is unclear whether iconicity is psychologically active when people actually use these words. Since these words have established meanings, speakers can produce and interpret them without needing to engage with their iconic qualities (Keller, 1998), relying instead on convention to understand them—essentially treating them as arbitrary. According to this view, although iconicity may have been salient when words like *gooey* and *puffy* were first coined, any appearance of iconicity in their current form is just an artifact of their creation, not a quality that is active for speakers when they typically use these words. From this perspective, decontextualized ratings of iconicity for English words may merely be metalinguistic judgments that do not correspond to depiction in language use.

In this study, we tested whether iconicity is active when English speakers use iconic words by conducting a quantitative study of gesture rates for independently rated high iconicity and low iconicity words within a large video corpus of American television news and interviews. We find, overall, that high iconicity words are more likely to co‐occur with iconic gestures. This association is largely independent of how much the word's meaning relates specifically to action and the senses, but it is not independent of the word's lexical category: high iconicity verbs are much more likely to co‐occur with iconic gestures than low iconicity verbs, while there is little difference between high and low iconicity adjectives. These results suggest that iconicity in spoken language may be most active, and manifest most strongly across modalities, when the meaning being expressed is broadly dynamic in quality, irrespective of its particular sensorimotor content.

## 2 Background

### 2.1 Iconicity in spoken words

Historically, linguists have disregarded iconicity in favor of the Saussurean principle that the linguistic sign is arbitrary (de Saussure, 1983; Hockett, 1959, 1978; Locke, 1689; Pinker, 1999; Pinker & Bloom, 1990; Whitney, 1867). However, in large part because of a far better understanding of sign languages, recent research has established that there is more iconicity in language than previously understood (Perniss et al., 2010). This includes increasing recognition of iconicity in spoken vocabularies (Dingemanse, Blasi, Lupyan, Christiansen, & Monaghan, 2015; Sidhu, 2025), which goes far beyond the iconic representation of sounds through onomatopoeia that is found across all spoken languages. For example, cross‐linguistic statistical analyses of vocabulary show evidence for iconic associations across a range of semantic domains including color terms (Johansson, Anikin, & Aseyev, 2020), touch‐related words (Winter, Sóskuthy, Perlman, & Dingemanse, 2022), size words (Haynie, Bowern, & LaPalombara, 2014; Winter & Perlman, 2021b), and shape‐related words (Sidhu et al., 2021), as well as a large number of basic vocabulary items (Blasi, Wichmann, Hammarström, Stadler, & Christiansen, 2016; Johansson, Anikin, Carling, & Holmer, 2020; Joo, 2020).

Cross‐linguistic research has also shown that the great majority of spoken languages feature a lexical class of ideophones (Voeltz & Kilian‐Hatz, 2001), which are often perceived as iconic—at least to native speakers. These words form a distinct lexical class of “marked words that depict sensory imagery” (Dingemanse, 2013, p. 143). For example, the Japanese ideophones *goro* and *koro* are both used to represent an object rolling (Vigliocco & Kita, 2006), with the repetition of /o/ depicting the rolling movement, especially in the reduplicated forms *gorogoro* and *korokoro*. In these ideophones, the voicing contrast reflects a difference in the weight and size of the rolling object: the voiced *goro* refers to movement of a heavy object, the voiceless *koro* to a light object. Through such iconicity, ideophones are used to convey rich sensory meanings. By their nature, these words extend beyond mere *descriptions* of sensory experience and become *depictions* of sensory experience, as in this case, a spoken enactment of a rolling motion. This is why Dingemanse (2011, p. 299) has described ideophones as “the next best thing to having been there.”

In comparison to many other spoken languages, some researchers have suggested that English and other Indo‐European languages are unusually lacking in iconicity. Perniss et al. (2010, p. 1) reasoned that, “if we look at the lexicon of English (or that of other Indo‐European languages), we might be forgiven for thinking that there could be anything but a conventionally determined, arbitrary connection between a given word and its referent.” More specifically, it has been asserted that English is distinctly lacking in ideophones (Diffloth, 1972; Kita, 1997; Liberman, 1975; Nuckolls, 2004, 2004). Such claims appear to place English as a lower‐bound case of how much iconicity might be expected in a spoken language. However, some linguists have challenged this view over the years, highlighting the iconic properties of English in both its lexicon and prosody (Bolinger, 1949; Bolinger & Sears, 1981; Waugh, 1992, 1994). Building on this, recent studies of iconicity ratings (reviewed in Winter & Perlman, 2021a) show that native speakers deem many common English words as iconic (Perry, Perlman, & Lupyan, 2015; Winter, Perlman, Perry, & Lupyan, 2023, 2017).

Among other methods, iconicity ratings—collected by asking native speakers to rate words on a continuous scale for how much they sound like what they mean—provide a way of operationalizing how more or less iconic a particular word is perceived to be by language users (Motamedi, Little, Nielsen, & Sulik, 2019; Vinson, Cormier, Denmark, Schembri, & Vigliocco, 2008; Winter & Perlman, 2021a). The by‐word averages that result from iconicity judgments enable researchers to compare how iconicity is distributed across different domains of vocabulary within a language, revealing which kinds of words more easily afford iconicity. Using iconicity ratings of English words, Winter et al. (2017) found that iconicity was higher for words relating to the senses than those with more abstract meanings, and that within highly sensory words, iconicity was higher for words relating to sound and touch compared to those relating to vision, smell, and taste. It stands to reason that auditory words would have high iconicity ratings as, intuitively, auditory experiences are more easily depicted within the spoken‐auditory channel. The high iconicity ratings for haptic words may be explained by the fact that haptic experiences are dynamic and require movement (Bartley, 1953; Carlson, 2010), and thus, the meanings of many haptic words may readily afford iconicity via mapping to the dynamic, temporal qualities of speech.

Further, Winter et al. (2017) found that iconicity ratings were highest for interjections, followed by verbs and adjectives, and finally nouns and grammatical words; as such, they were inversely correlated with frequency. The high iconicity ratings for interjections could be due to their relationship with auditory experiences: some interjections, such as *bang* or *pow* are onomatopoeic, while others, such as *ow* or *ugh*, resemble the vocalizations that are often made when experiencing pain or disgust. The higher iconicity ratings for verbs than nouns may be explained by the fact that verbs often represent more dynamic, movement‐based concepts than nouns, which have more static, property‐based meanings (Givón, 1984; Langacker, 2008; Strik Lievers & Winter, 2018).

However, the use of iconicity ratings as a method of operationalizing iconicity has been criticized, and their construct validity has been questioned. Thompson et al. (2020) suggested that these ratings may not be based on iconicity per se, but on other aspects of a word, such as whether it is structurally similar to onomatopoeias or the degree to which a word's meaning is related to the senses. Indeed, iconicity ratings are ultimately metalinguistic judgments that happen in a decontextualized environment: it may be possible that the iconicity that English speakers see in a word judgment task does not correspond to a psychologically active form of iconicity in actual language use.

### 2.2 Iconicity in gesture

Whereas modern linguistics has been slow to recognize the iconicity of many spoken words, the iconicity of many gestures has been much more clearly apparent. Iconic gestures are those that resemble what they represent, often a physical thing or action (McNeill, 1992, p. 12). For example, while describing an object being squished, a speaker might mime squishing the object between two hands by moving them, palms facing each other, inward. Iconic gestures can also represent more abstract concepts. For example, when comparing two things, a speaker might hold each open hand out in front of them with their palms facing upward, and move them up and down interchangeably, to resemble the movement of a pair of weighing scales.

Multimodal approaches to language consider speech and co‐speech gesture to be integral parts of the same utterance (De Ruiter, 2000, 2007; Enfield, 2009; Kendon, 2004; McNeill, 1992, 2005, 2015). This approach holds that both speech and gestures are produced by the same cognitive process (De Ruiter, 2000), and some gesture theorists have posited a “growth point,” an initial minimal unit of thought that is neither solely linguistic or imagistic, from which both speech and gesture develop within a speaker's utterance (McNeill, 1992, 2015; McNeill & Duncan, 2000). As a result, the gesture is driven by meaning that does not correspond directly with individual words but instead larger linguistic units, sometimes called “conceptual affiliates” (De Ruiter, 2000, p. 291). For example, in McNeill's (1992, p. 112) study of gestures participants used when recounting a Looney Tunes cartoon, a speaker said, “when he gets up to the bird the grandma hits him over the head with the umbrella,” and used a hitting gesture in time with the entire verb phrase “grandma hits him.” Here, the imagistic side of the growth point, the mental image of a hitting action, forms the gestural part of the utterance, whereas the conceptual affiliate is reflected in the categorical content of the event, including the agent (“grandma”) and patient (“him”). The idea of a growth point understands speech and gesture to be two sides of a dynamic and integrated system of communication where imagistic and linguistic aspects work together as acts of meaning‐making emerging from the same conceptual source.

There are several factors that may motivate the production of iconic gestures during spoken language use. One factor is the semantics of the utterance. It has been argued that speakers use iconic gestures to express aspects of visuospatial and motor imagery (Hostetter & Alibali, 2019; Kita, Alibali, & Chu, 2017). Hostetter and Alibali (2008, 2019), in their Gesture as Simulated Action (GSA) framework, proposed that iconic gestures arise as a product of underlying embodied simulations of actions and perceptual states that are generated as a part of thinking‐for‐speaking. In support of this hypothesis, it has been found that people gesture more frequently when communicating about topics related to motor or visual imagery than those which were more abstract (Feyereisen & Havard, 1999). The GSA framework would also suggest that verbs, which represent more dynamic concepts, would be more strongly associated with the production of iconic gestures than nouns and adjectives, which tend to have more static meanings (Givón, 1984; Langacker, 2008; Strik Lievers & Winter, 2018).

Additionally, studies of how people use ideophones suggest that iconicity in the words of speech may also be an important factor in the production of iconic gestures. Linguists have documented a tight relationship between ideophones and gestures across speakers of different languages (Diffloth, 1972; Dingemanse, 2013; Kita, 1993; Kunene, 1965; Zondo, 1982). The gestures that speakers produce with ideophones are typically iconic, depicting the same concept represented by the ideophone (Dingemanse, 2013; Dingemanse & Akita, 2017; Kita, 1997). Together, Dingemanse argues, the ideophone and iconic gesture are “two aspects of the same process of depiction” (Dingemanse, 2013, p. 143). When using ideophones, “the speaker actually turns actor and performs the action referred to” (Kunene, 1965, p. 36).

The production of iconic gestures is also associated with the use of spoken quotations (Blackwell, Perlman, & Fox Tree, 2015). Quotations are grammatical constructions speakers use to report events, typically by depicting rather than describing them. These constructions use quotative verbs like *say*, *be like*, or *go* to introduce quoted content, which is typically a person's speech, but also commonly includes various other kinds of actions and events (Clark & Gerrig, 1990). Quotations are often multimodal, involving depiction with both the voice and body (Blackwell et al., 2015). For example, when reporting a child saying “Daddy pass the ketchup,” a speaker might mimic both the high‐pitched voice as well as the upward gaze of the child, as if looking up at their father. Comparing the use of depiction across different quotations produced by English speakers, Blackwell et al. found that the level of depiction in the voice and body were correlated, supporting the hypothesis that, in quotation, the vocal and bodily channels are integrated together to form a unified, multimodal depiction arising from one central concept.

Notably, it has been reported that ideophones are often used within quotation‐like constructions (Childs, 1995, p. 187), where they serve to depict sensory aspects of the quoted context. In this role, ideophones are often marked for their function as depictive words by being syntactically isolated from other words in the utterance (Dingemanse, 2012, 2013; Dingemanse & Akita, 2017; Kruspe, 2004; Nuckolls, 1996). In a study of the morphosyntactic behavior of Japanese ideophones, Dingemanse and Akita (2017) found that when these words were more syntactically isolated within an utterance, they tended to be used with more expressive intonation, phonation, and morphology, and were also more likely to be used with iconic gestures (Dingemanse & Akita, 2017).

Finally, the frequency of words or phrases may be a relevant factor for iconic gesture production. Using TV news data to study the co‐occurrence of temporal expressions and iconic gestures related to time, Pagán Cánovas, Valenzuela, Alcaraz Carrión, Olza, and Ramscar (2020) found that infrequent expressions were more likely to co‐occur with gestures. This finding was replicated by Alcaraz‐Carrión, Alibali, and Valenzuela (2022) when studying expressions regarding numerical concepts and semantically related iconic gestures.

### 2.3 Current study

In the current study, we use the TV News Archive, a large multimodal corpus of television news and interviews, to compare how frequently English speakers produce iconic gestures when using words rated as high and low in iconicity. Under a division‐of‐labor view where speech primarily functions for arbitrary description and gesture for depiction, it would not necessarily be expected that iconic gestures would go together with iconic words. On this view, the iconicity of words is not typically active for speakers. If, on the other hand, depiction is a fundamentally multimodal process, we would expect that the use of highly iconic words would reflect an underlying multimodal depiction that manifests in iconic gestures as well as iconic speech.

As we operationalize iconicity in English words via iconicity ratings, our analysis speaks to methodological debates about the construct validity of these ratings. The finding that words rated high in iconicity in an independently conducted study also tend to co‐occur with spontaneously produced iconic gestures would present new evidence that the results of lexical rating tasks do indeed correspond to a psychologically active form of iconicity. Considering evidence that gestures correspond with conceptual affiliates rather than individual words, the finding that word‐level iconicity is predictive of the occurrence of iconic gestures in utterances would be all the more remarkable.

As we have seen above, factors other than a word's iconicity may also contribute to the propensity of a speaker to gesture, including its frequency, relationship to visuospatial and motor imagery, and morphosyntactic behavior. There is potentially a complex relationship between these variables. For example, previous research has found an inverse relationship between frequency and gesture, and also that people gesture more when talking about topics that involve motor and visual imagery. Research on iconicity in words has found iconicity is inversely correlated with frequency and positively correlated with the degree to which a word's meaning relates to the senses. Thus, in this study, we investigate the relation between iconic words and iconic gestures while also considering words’ frequency and sensorimotor ratings, as well as their part‐of‐speech and morphosyntactic behavior within utterances.

## 3 Materials and methods

All code and analyses can be found in the following OSF repository: https://osf.io/3myx4/.

### 3.1 TV News Archive

Our data come from The TV News Archive, an open‐access database of 2.5 million U.S. TV news broadcasts (https://archive.org/details/tv). This archive offers two specific benefits for gesture research (Winter, Perlman, & Matlock, 2013; Woodin, Winter, Perlman, Littlemore, & Matlock, 2020). First, it allows researchers to search specific words or phrases to easily identify relevant naturally occurring multimodal data, which can otherwise be difficult to obtain for gesture research. Second, researchers can collect a large sample of gestures produced by hundreds of different speakers, allowing for quantitative analysis that is not often possible in other methods of data collection.

### 3.2 Search term selection

We operationalized iconicity by selecting high and low iconicity search terms using the subjective ratings from Winter et al. (2023). The iconicity ratings were collected for more than 14,000 English words by asking American English speakers to rate each word for how much it “sounds like” what it means on a scale of 1 (not iconic at all) to 7 (very iconic). Each word was rated by at least 10 participants. We selected 24 search terms rated high in iconicity (each with an average rating of at least 6.3) and 24 words rated low in iconicity (each with an average rating of at most 2.4).

Table 1 shows the 48 search terms used in our study along with their average iconicity ratings.

**Table 1:** The search terms used in the study

Iconicity type	Search term	No. of eligible tokens	Iconicity rating (1‐7)	Dominant modality	Max. perceptual strength (1‐5)	Dominant action effector	Max. action strength (1‐5)	Dominant POS	Frequency (per million words)	Iconic gesture rate	Non‐iconic gesture rate
Low iconicity	ordain	21	1.5	visual	2.7	mouth	2.7	verb	0.18	0%	76%
	knew	54	1.8	interoceptive	2.8	head	3.7	verb	368.96	7%	52%
	other	50	1.8	visual	2.6	head	1.2	adj.	735.39	18%	48%
	rejoin	34	1.9	visual	2.6	head	2.3	verb	1.25	6%	44%
	filling	51	2.0	visual	2.8	mouth	1.9	verb	9.69	14%	65%
	realize	58	2.0	interoceptive	2.5	head	3.9	verb	79.06	7%	52%
	grateful	43	2.1	interoceptive	4.2	head	3.5	adj.	26.57	5%	23%
	put	35	2.1	visual	3.2	hand/arm	4.1	verb	828.45	43%	34%
	barren	3	2.1	visual	3.4	head	1.5	adj.	1.47	33%	33%
	confirmed	34	2.1	visual	2.8	mouth	2.9	verb	13.51	6%	41%
	exact	46	2.1	visual	3.8	head	2.0	adj.	22.63	22%	61%
	jealous	30	2.1	interoceptive	3.3	head	3.2	adj.	38.27	10%	37%
	wearing	56	2.1	visual	3.8	foot/leg	4.3	verb	86.47	5%	66%
	acquaint	8	2.2	visual	3.3	head	1.9	verb	0.39	0%	63%
	inform	57	2.2	auditory	3.5	mouth	3.0	verb	12.63	11%	5%
	outwit	14	2.2	visual	2.5	head	3.1	verb	0.59	7%	50%
	covet	22	2.2	interoceptive	2.6	head	3.1	verb	0.94	9%	55%
	prevail	40	2.2	interoceptive	2.6	head	2.4	verb	2.1	0%	35%
	sullen	11	2.2	visual	2.9	head	2.9	adj.	0.57	0%	45%
	ate	0	2.3	gustatory	3.2	mouth	4.6	verb	33.76	N/A	N/A
	absent	13	2.3	visual	3.2	head	2.4	adj.	2.57	0%	8%
	said	303	2.3	auditory	4.6	mouth	4.9	verb	1108.45	5%	32%
	discern	32	2.4	visual	3.1	head	3.0	verb	0.33	22%	56%
	tamper	36	2.4	visual	3.2	hand/arm	2.2	verb	0.49	22%	42%
High iconicity	gooey	1	6.3	haptic	4.2	hand/arm	3.4	adj.	0.76	0%	0%
	slushy	67	6.3	visual	3.1	mouth	2.8	adj.	0.06	12%	61%
	spank	97	6.3	haptic	4.0	hand/arm	4.6	verb	3.39	12%	57%
	crumbly	0	6.3	haptic	3.1	hand/arm	1.8	adj.	0.12	N/A	N/A
	barking	11	6.4	auditory	5.0	head	3.3	verb	9	0%	64%
	chomp	14	6.4	visual	2.9	mouth	4.7	verb	0.25	36%	57%
	snap	2	6.4	auditory	3.7	hand/arm	3.5	verb	17.39	0%	100%
	wring	21	6.4	haptic	3.5	hand/arm	4.1	verb	1.22	52%	29%
	zap	15	6.4	haptic	2.8	hand/arm	2.4	verb	2.45	33%	53%
	plump	5	6.4	visual	3.1	head	2.1	adj.	1.47	0%	80%
	saggy	17	6.5	visual	3.9	head	1.9	adj.	0.57	0%	35%
	bobbing	0	6.5	visual	3.8	head	3.2	verb	0.61	N/A	N/A
	crispy	14	6.5	haptic	3.4	mouth	3.2	adj.	2.27	7%	64%
	yucky	56	6.5	gustatory	3.0	mouth	3.5	adj.	0.43	4%	43%
	munch	5	6.6	gustatory	2.7	mouth	4.3	verb	0.84	20%	40%
	splotch	36	6.7	visual	4.4	head	2.2	verb	0.08	28%	67%
	puffy	24	6.7	visual	4.1	head	2.5	adj.	1.69	8%	46%
	squish	37	6.7	haptic	3.5	hand/arm	3.1	verb	1.04	81%	8%
	wobbly	5	6.7	visual	3.4	foot/leg	3.2	adj.	0.73	60%	20%
	bang	6	6.8	auditory	4.1	head	2.8	verb	19.98	83%	17%
	woof	13	6.8	auditory	4.7	head	2.5	verb	2.76	38%	23%
	wheeze	13	6.9	auditory	4.2	mouth	3.6	verb	0.57	8%	31%
	swish	21	6.9	visual	3.3	head	1.9	verb	1.61	29%	43%
	swoosh	15	7.0	auditory	3.4	hand/arm	1.5	verb	0.24	13%	53%

These 48 search terms were balanced, as much as possible, for perceptual strength, part‐of‐speech, and syllable length. To ensure both word lists were comparatively sensory, we used perceptual strength ratings from the Lancaster Sensorimotor Norms (Lynott, Connell, Brysbaert, Brand, & Carney, 2020). This is a database of 39,707 concepts with perceptual strength ratings for six modalities—the five “folk” senses (sight, sound, smell, touch, and taste) and interoception (the internal state of the body, including hunger, pain, and temperature)—and action strength ratings for five action effectors—foot/leg, hand/arm, head excluding mouth, mouth/throat, and torso. Words that did not appear in the Lancaster Sensorimotor Norms were not included in the list of search terms.

Table 1 shows each word's dominant modality, maximum perceptual strength rating, dominant action effector, and maximum action strength rating. The dominant modality and action effector refer to the sense and body part with the highest average perceptual and action strength rating. The maximum perceptual and action strength ratings correspond, respectively, to the highest rated perceptual modality and action effectors. When selecting the low iconicity words, we set a maximum perceptual strength rating of at least 2.5 to match the high iconicity words. However, because English words relating to sound and touch tend to be more iconic than those relating to vision, taste, and smell (Winter et al., 2017), we could not balance the word lists for dominant sensory modality without increasing the iconicity rating of our “low iconicity” words and decreasing the average iconicity rating of our “high iconicity” words. This means that high and low iconicity words are not equally represented across all modalities—for instance, there are no dominantly interoceptive high iconicity search terms. Likewise, the word lists were not balanced for dominant action effector—hence, for example, there are many more hand/arm words that are high in iconicity than low in iconicity.

The word lists were also balanced for part‐of‐speech. All search terms were identified as being at least 70% dominantly adjectives or verbs using the SUBTLEX part‐of‐speech frequencies (Brysbaert, New, & Keuleers, 2012), a corpus of U.S. film and TV subtitles containing 51 million word tokens from over 8000 subtitle files. While this measure of part‐of‐speech (POS) reflects the most common usage of words within the particular genre of U.S. film and TV, the words as used in any particular instance may occur as different parts‐of‐speech. For example, the word *bang* is defined as a verb using the SUBTLEX frequencies, but is sometimes used as an interjection in the videos we analyze. Given the time it would take to transcribe each video and code the search term for part‐of‐speech, we opted to rely on the SUBTLEX frequencies to classify the words according to their most frequent POS. However, as described further below, we also investigated the morphosyntactic behavior of a subset of the search terms according to their particular use within the videos (see Section 5).

Finally, we also balanced the word lists, as much as possible, for syllable length. There is a marked difference in the average length of the high and low iconicity search terms—the low iconicity search terms were on average longer than the high iconicity terms. To limit this, we set a maximum word length of two syllables for the low iconicity words. This resulted in 14 one‐syllable and 10 two‐syllable high iconicity words, and 4 one‐syllable and 20 two‐syllable low iconicity words.

**Fig. 1:**
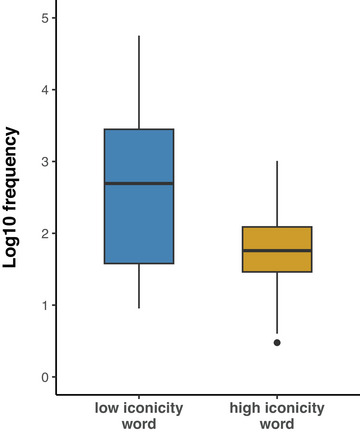
Bar plot of log SUBTLEX frequency (Brysbaert & New, 2009) of words rated to be low (left) and high (right) in the iconicity ratings from Winter et al. (2023).

We were not able to balance the word lists for word frequency because high iconicity words are generally less frequent than low iconicity words (Lupyan & Winter, 2018; Perry et al., 2015; Winter et al., 2023). Table 1 shows each word's frequency per million words taken from SUBTLEX word frequencies (Brysbaert & New, 2009). Here, we can see, for example, that the low iconicity word *said* had the highest frequency at 1108.45 tokens per million words, while *slushy* has the lowest frequency at 0.06 tokens per million words. As shown in Fig. 1, there is a large difference (Cohen's *d* = 0.91) between the log frequencies of the high iconicity (average frequency of 2.9) and low iconicity words (average frequency of 140.61). This difference is notable because of the finding that people gesture more alongside phrases which are less frequent (Alcaraz‐Carrión et al., 2022; Pagán Cánovas et al., 2020). If word frequency is inversely correlated with gesture rate, the high iconicity search terms, which are much less frequent, could be expected to co‐occur with gestures more often due to their low frequency, regardless of their iconicity rating. We addressed this possible confound by including word frequency as a covariate (fixed effect) in our statistical models.

### 3.3 The dataset and data filtering

We began creating the dataset by downloading a list of 100 video URLs per search term from the TV News Archive, resulting in 4800 videos in total. In many videos, the search term was used more than once, so each instance was coded as a separate token. This resulted in a total of 5725 tokens. We then filtered the tokens according to the criteria below. The remaining tokens were considered “eligible” for gesturing. Data from the TV News Archive often does not allow for gesture analysis, such as, for example, when a speaker is not shown, or their hands are obscured, for example, by news captions. Thus, it is common for gesture studies using the TV News Archive to have a large number of tokens that cannot be analyzed (cf. Woodin et al., 2020). Here, we detail our data filtering process and the steps we undertook to arrive at a dataset amenable to gesture analysis.

#### 3.3.1 Was the search term used?

We removed tokens where either the search term was not used, was used with a derivational morpheme, or was used as a proper noun (*n* = 1921, 34% of total tokens). This included tokens where there was a transcription error, or the search term referred to a brand name (e.g., Nike swoosh or Slushie), or a person's name (e.g., the nickname Swoosh for a basketball player). The TV News Archive search function searches for the lemma rather than the precise word, so, for instance, when searching the word *wobbly*, it also includes results for *wobble*, *wobbles*, *wobbled*, and so on. Tokens were filtered from the data when the search term had morphological variation, which was derivational (such as *information* rather than *inform)*, but not if the morphological variation was inflectional, that is, modifying the grammatical form of the word, but not changing its part‐of‐speech (such as *informs* from *inform*). However, there was some variation in the part‐of‐speech of each search term in the context of the videos. For example, the word *discerning* (defined as a verb in our data) was used as both a present continuous verb and a deverbal adjective, but both contexts were included.

#### 3.3.2 Was the token a duplicate?

We discarded duplicate tokens (*n* = 682, 12% of total tokens) resulting from repeated broadcasts of the same footage.

#### 3.3.3 Was the speaker visible?

We excluded tokens where the speaker was not visible (*n* = 899, 16% of total tokens). Tokens were also discarded if the speaker was visible only from a distance such that it was difficult to identify them (e.g., in broadcasts of courtrooms).

#### 3.3.4 Were the speaker's hands visible?

We excluded tokens where both of the speaker's hands were not visible (*n* = 601, 10% of total tokens), for example, due to a close‐up camera angle. If the speaker's hands were partially obscured, but decisions could still be made for the following coding steps, then the token was not discarded. For example, if a speaker had their hands on a podium, but their wrists were still visible, making it clear that there were no gestures, then the token was not discarded. If at least one hand was visible, the token was not discarded. This allowed for as much viable data to be retained as possible.

#### 3.3.5 Were the speaker's hands free to gesture?

In the final step of data filtering, we excluded tokens where the speaker's hands were not free to gesture (*n* = 76, 1% of total tokens). This proved difficult to determine, as in most tokens where a speaker was holding something, it is reasonable to assume that they could have either gestured while holding the object or made their hands available to gesture. For example, in some tokens, the speaker was holding a presentation clicker but could still gesture. If a speaker's hands were occupied by something that they were actively using, such as reading from a piece of paper, then the token was discarded. However, if the speaker was not using the object in their hand (such as holding a pen with which they were not writing), then their hands were deemed free to gesture. If one hand was occupied but the other was not, the token was not discarded.

### 3.4 Gesture coding

After filtering the data, we were left with 1546 tokens that could be analyzed for gesture. Although we had to exclude a total of 4179 tokens (73% of the data), the remaining dataset was still relatively large for gesture analysis.

There were more eligible tokens for low iconicity (1051) than for high iconicity words (495). This discrepancy resulted, in part, from more frequent repetition of the low iconicity words in the clips, likely reflecting their generally higher frequency. The word *said* was particularly over‐represented, with 303 eligible tokens. The words with the second and third highest number of eligible tokens were *spank* (*n* = 97) and *slushy* (*n* = 67). The words with the fewest eligible tokens were *barren* (*n* = 3), *snap* (*n* = 2), and *gooey* (*n* = 1), while the words *ate*, *bobbing*, and *crumbly* did not have any tokens eligible for gesture analysis after the data filtering steps explained in Section 3.3. In total, we were left with 45 search terms in our gesture analyses: 22 high in iconicity and 23 low in iconicity.

#### 3.4.1 Did the speaker gesture?

The eligible tokens (*n* = 1546) were then coded for whether the speaker gestured during the production of the search term. Although gestures can be produced with many parts of the body, for the purposes of this study, only manual gestures, that is, gestures made with the hands, were coded. Gestures differ from other body movements by virtue of being understood as purposefully communicative (Kendon, 2004, Chapter 2). Thus, instances of fidgeting, such as playing with hair or adjusting glasses, were not considered gestures.

Our analysis focused on gestures that were produced in co‐occurrence with the articulation of the targeted search terms, that is, manual gesture production (partially) overlapped with the production of the search term. We defined co‐occurrence as when the word was produced in overlap with the pre‐stroke, stroke, or post‐stroke hold of the gestures. If the search term was spoken as the speaker moved their hands to a resting position, then the gesture was not coded as having co‐occurred with the search term.

#### 3.4.2 Context

Tokens in which a gesture occurred were also coded for context to better understand what proportion of gestures came from “natural” spontaneous speech, and what proportion were scripted. The contextual categories emerged inductively through coding the tokens, and broadly fit into a spectrum from most natural to most scripted. The categories were: unscripted interview or conversation; presenting to camera; presenting in front of a screen; giving a speech, lecture, presentation, press conference, or Q&A; speaking in court, senate, or congress; semi‐scripted interview or conversation, including teleshopping; scripted acting in an advert, sitcom, or music video.

#### 3.4.3 Was the gesture iconic?

**Fig. 2:**
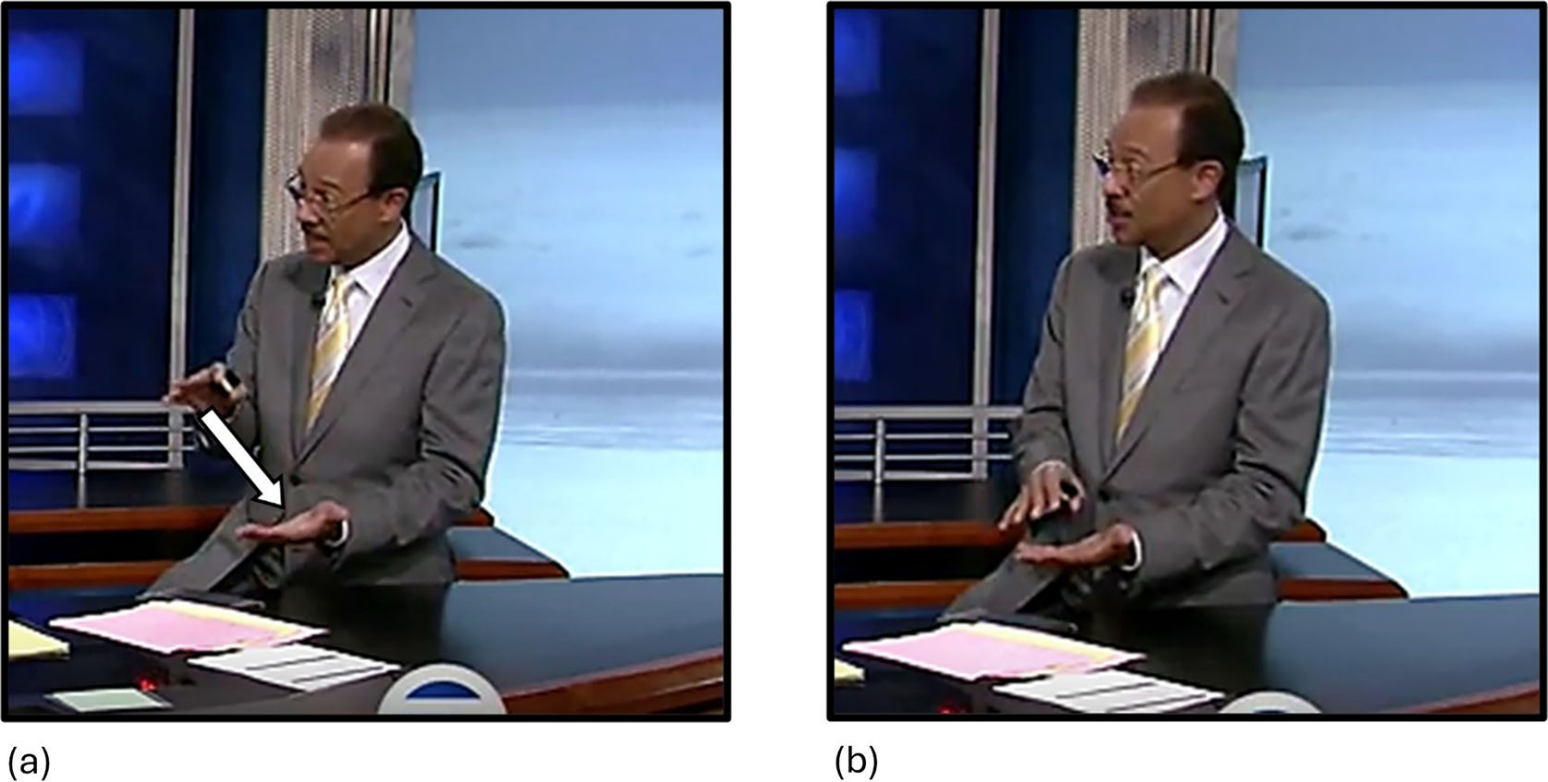
Example of a biphasic iconic enactment gesture. (a) “it's ***getting*** …” The speaker's open hands are held apart, palms facing each other. His left hand is at hip level, palm facing upward, and his right hand is at chest level, palm facing downward. The superimposed arrow indicates the path of movement. (b) “… ***squished*** now.” The speaker moves his right hand down toward his left hand in two strokes. It lands slightly above his left hand, at around hip level. Bold italics indicate speech that co‐occurs with the stroke phase of the respective gesture.

Gestures (*n* = 882) were then coded for whether they were iconic, that is, whether they visibly reflected some aspect of the conceptual content of the utterance. Iconic gestures were identified if they appeared to use any of the following (not mutually exclusive) strategies: modeling, enactment, depiction, and metaphor. The first three strategies are Kendon's (2004) types of representational (i.e., iconic) gestures. In enactment, a speaker mimed performing an action, such as in Fig. 2, where the speaker said the word *squish* while enacting squishing something between two hands, with an open palm moving down toward another open palm. When modeling, a speaker's hand formed the shape of what was being referred to, for example, while saying the word *chomp*, one speaker in our dataset repeatedly moved their fingers and thumbs on one hand up and down to resemble a mouth biting down on something. The difference between modeling and enacting can also be characterized in terms of observer versus character viewpoint (McNeill, 1992), that is, whether the speaker is representing an action as if they were observing it, or performing it themselves. When depicting, a speaker's hands traced the shape of something in the air, for example, another speaker in our dataset said the word *puffy* to describe a coat while moving their hands outward from their body in an arc, to resemble the shape of a puffy coat.

**Fig. 3:**
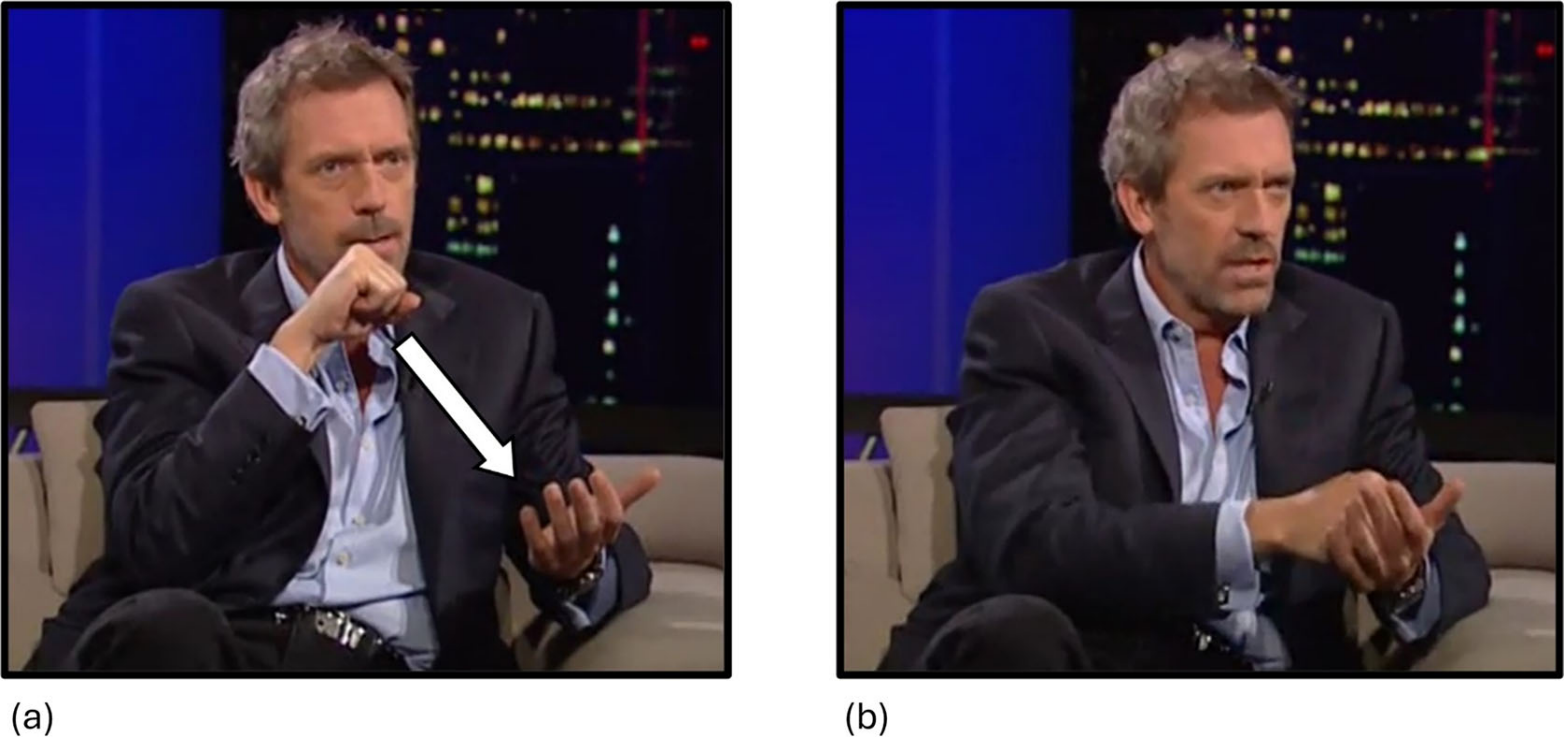
Example of a metaphorical iconic gesture. (a) “the audience was something to be…” The speaker's right hand starts at shoulder level in a fist, palm facing downward. His left hand is at stomach level, open, palm facing upward. The superimposed arrow indicates the path of movement. (b) “… ***outwitted***.” His right hand, in a fist, moves downward and lands in his left hand, which remains open. Bold italics indicate speech that co‐occurs with the stroke phase of the respective gesture.

The fourth iconic gesture type were metaphoric gestures, that is, those that represented one thing in terms of another, even if both were physical/concrete (as in Cienki & Müller, 2008). The speaker could use the same or different metaphors in speech and in gestures, or the metaphor could be expressed in a gesture but not in speech (Cienki & Müller, 2008; Winter et al., 2013). For example, in Fig. 3, while saying the low iconicity word *outwit*, a speaker performed a gesture where they brought a fist down suddenly on an open flat palm. This was coded as an iconic gesture, as the speaker enacted pounding something with his fist. But, as the search term *outwit* is not literally synonymous with the act of pounding, it was also coded as metaphoric, with the pounding action metaphorically representing the idea of beating someone (by outwitting them).

The gestures that were not coded as iconic generally included beat and deictic gestures. Beat gestures punctuate speech and provide emphasis (McNeill, 1992, p. 15), for example, a speaker might make repeated strokes with their hand in time with key words or moments of prosodic prominence. Deictic gestures are those that point to something, often but not always with an index finger (McNeill, 1992, p. 18). The forms of these gestures did not clearly reflect any conceptual content of the utterance.

#### 3.4.4 Inter‐coder reliability

The full coding process was performed by a single coder (first author), who developed a coding handbook detailing the above steps. During the coding process, they identified common problems among difficult‐to‐code tokens, which were discussed by all authors until an agreement was reached on how the tokens should be coded, and the coding handbook was updated to reflect this. The coder then iteratively coded tokens that were marked as “borderline” cases. To check for inter‐coder reliability, a second coder, naïve to the purpose of the study, was given a random 25% sample (*n* = 388) of tokens that were coded as “eligible” for gestures, balanced for the words’ iconicity type (194 high iconicity tokens, 194 low iconicity tokens). The data were presented without the words’ iconicity type listed, and in random order. Trained on the coding handbook, the second coder coded for whether a gesture occurred and whether the gesture appeared to be iconic. The results of this check revealed general agreement between the two coders: 89% for whether a gesture occurred and 84% for whether the gesture was iconic (only considering tokens where both coders agreed a gesture had occurred). Using Cohen's kappa, both the gesture coding (*k* = 0.77) and iconic gesture coding (*k* = 0.63) showed substantial agreement.

### 3.5 Statistical analysis

We used R version 4.1.1 (R Core Team, 2019) and the tidyverse package version 1.3.2 (Wickham et al., 2019) for data analysis. The patchwork package version 1.1.1 (Pederson, 2020) was used for creating multi‐plot arrays. The brms package version 2.16.2 (Bürkner, 2017) was used for Bayesian models.

We fitted three Bayesian logistic regressions to specifically test the hypothesis that high iconicity words are associated with more gestures. The first model was fitted on the overall count of gestures, which meant that this model estimated the proportion of tokens without any gestures versus tokens with any gestures. The second model specifically looked at only those gestures that were coded as iconic. The final model only looked at the count of gestures that are not iconic. The comparison of this “other gestures” model to the “iconic gestures” and “any gestures” model allowed us to assess the extent to which high iconicity words are associated with an overall higher propensity to gesture, or instead with a propensity to specifically use iconic gestures.

All models had the same structure, with a fixed effect of “word type” (high iconicity vs. low iconicity). We added random intercepts for “word” and “video,” as we have multiple tokens for each word, as well as multiple tokens for each video. No by‐type random slopes were used for these random intercepts since “type” does not vary within word (a word is either high iconicity or not), nor does it vary within video (each video is focused on one word). Our models also included word frequency as a covariate (fixed effect) to see whether iconicity “word type” had an effect on gesture rate beyond the fact that high iconicity words are also less frequent.

We used Student's *t* distribution with df = 3, mean = 0, and scale = 2.5 as priors for the intercept and standard deviation of the model, and following the recommendations by Gelman, Jakulin, Pittau, and Su (2008), we used a weakly informative Cauchy prior for the “type” effect with center 0 and scale 2.5. Models were estimated using Markov Chain Monte Carlo Simulation (2000 post‐warm‐up samples × 4 chains), and convergence was adequate (all Rhat = 1.0, all ESS > 1000). Posterior predictive simulations showed that data simulated from the model looked similar to the actual data distribution.

## 4 Results

### 4.1 Descriptive statistics

Of 1546 eligible tokens, there were 881 gestures, 212 of which were iconic. Disregarding the word's iconicity type, the overall proportion of tokens where speakers produced a gesture was 62%. Of these gestures, 24% were coded as iconic. Table 2 shows the average gesture rates for all gestures, iconic gestures, and other gestures. When separating the data by the words’ iconicity type, we found that speakers gestured more when using high iconicity words than low iconicity words. Low iconicity words were produced with a gesture 56% of the time, while high iconicity words had a gesture proportion of 69%. This difference was most pronounced for iconic gestures: low iconicity words were produced with an iconic gesture 11% of the time, while high iconicity words had a proportion more than twice as high, at 24%. For other gestures (i.e., noniconic gestures), low iconicity words had a gesture proportion of 44%, while high iconicity words had a gesture proportion of 45%. We can use Cohen's *d* as a standardized measure of effect size to compare how strongly high iconicity words differ from low iconicity words for the different types of gesture. This measure reveals a medium effect size for the overall gesture proportion (*d* = 0.61) and the proportion of iconic gestures (*d* = 0.66), in contrast to a negligible effect size for the noniconic gestures (*d* = 0.027). As the overall gesture proportion also includes iconic gestures, these comparisons show that it is specifically iconic gestures that are more frequent when utterances contain high iconicity as opposed to low iconicity words.

**Table 2:** Proportion of gestures out of the total number of eligible tokens, averaged by word‐level iconicity

Word‐level iconicity	No. of words	All gestures	Iconic gestures	Noniconic gestures
**Low iconicity**	23	0.555	0.109	0.444
**High iconicity**	22	0.689	0.239	0.450

**Fig. 4:**
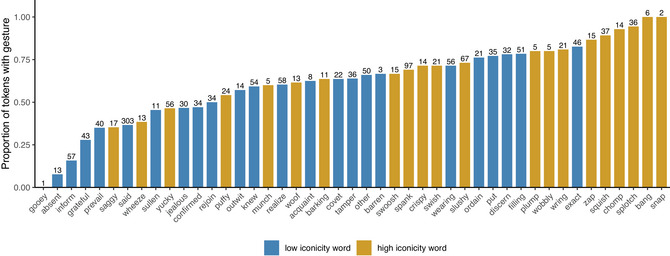
Proportion of tokens with gestures (iconic or not) for each word. The numbers at the top of each bar indicate the number of eligible tokens each proportion is based on. Colors indicate whether the word is high or low in iconicity.

Fig. 4 shows the proportion of gestures separately for each search term in our sample. Here, we can see that out of the 10 words with the highest proportions of gesture, only a single one, the word *exact*, belonged to the low iconicity category. Overall, most of the low iconicity words were associated with lower proportions of gestures, and thus appear toward the left of the graph. For the overall proportion of gestures, the average rank of high iconicity words was 9th, while for low iconicity words, it was 14th. This supports the finding that, on average, gestures more often co‐occurred with high iconicity words than low iconicity words.

**Fig. 5:**
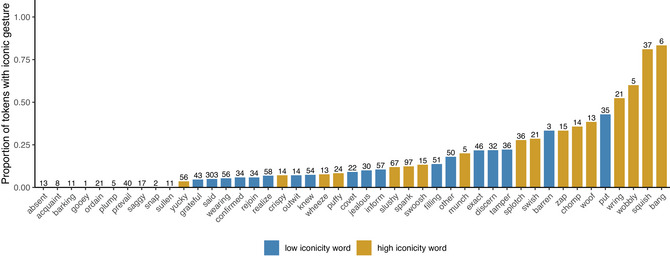
Proportion of tokens with iconic gestures for each word. Of the words with a proportion of 0, five were low iconicity (*absent*, *acquaint*, *ordain*, *prevail*, *sullen*), and five were high iconicity (*barking*, *gooey*, *plump*, *saggy*, *snap*).

Fig. 5 is similar to Fig. 4, but for iconic gestures only. Here, we see a similar pattern where out of the 10 words with the highest proportions of iconic gestures, only two were low in iconicity (*put* and *barren*). Most of the low iconicity words were associated with lower proportions of iconic gestures, and thus appear toward the left of the graph, although this difference is visually not as marked as in Fig. 4 for all gestures. For iconic gestures, the average rank of high iconicity words was 10th, while for low iconicity words, it was 13th. This supports the finding that, on average, iconic gestures more often co‐occurred with high iconicity words than low iconicity words.

**Fig. 6:**
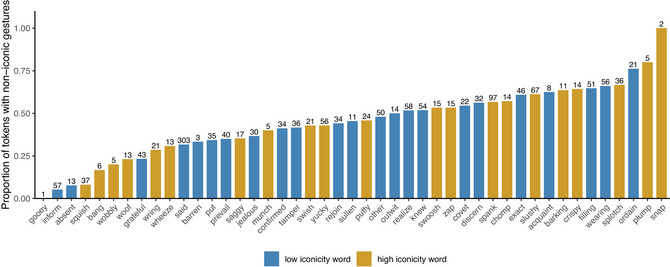
Proportion of tokens with noniconic gestures for each word.

Fig. 6 shows the proportion of noniconic gestures for each search term. Here, we see no clear pattern for word‐level iconicity: low iconicity and high iconicity words are quite evenly spread throughout the graph. Compared to Figs. 4 and 5, there were more low iconicity words with higher proportions of noniconic gestures, such as *ordain*, *wearing*, *filling*, *acquaint*, and *exact*. There were also many more high iconicity words with lower proportions of noniconic gestures, such as *squish*, *bang*, *wobbly*, and *woof*. For noniconic gestures, the average rank of high iconicity words was 11th, while for low iconicity words it was 12th. Comparison of all three figures suggests that the pattern of word‐level iconicity observed for all gestures (Fig. 4) is driven by iconic gestures (Fig. 5), and not by noniconic gestures (Fig. 6), such as beat gestures and deictic gestures.

Previous research using TV news data to study word‐ and phrase‐level predictors of gesture frequency found a correlation between less frequent expressions and higher gesture rates (Alcaraz‐Carrión et al., 2022; Pagán Cánovas et al., 2020). As frequency is inversely correlated with iconicity ratings (Perry et al., 2015; Winter et al., 2017), it was important to examine whether the difference between high and low iconicity words was actually due to a difference in iconicity, rather than word frequency. We assessed this descriptively by looking at the correlation between frequency and the proportion of gestures. Pearson's *r* did not suggest that average by‐word frequency was correlated with *any* of the three different types of gesture proportion we discussed above (proportion of any gesture, iconic gesture, or noniconic gesture). For the proportion of all gestures, the correlation coefficient was close to zero (*r* = −.08, *t*(43) = −0.52, *p* = .61). For the proportion of iconic gestures, the correlation with frequency was even lower (*r* = −.01, *t*(43) = −0.08, and *p* = .94). For noniconic gestures, the correlation with frequency was also low (*r* = −.08, *t*(43) = −0.52, and *p* = .61).

As discussed in Section 3.4.2, we also annotated the communicative context in which a token occurred, for example, whether it occurred in “natural,” spontaneous, versus more scripted speech. This revealed that most tokens where a gesture occurred were from unscripted interviews or conversations (*n* = 307, 35%), followed by speeches, lectures, presentations, press conferences, and Q&As (*n* = 195, 22%). The proportion of tokens produced with gestures was lower still for reports where the speaker was presenting to the camera without a screen behind them, such as a news anchor (*n* = 134, 15%) or presenting to the camera in front of a screen, such as a weather forecaster (*n* = 131, 15%). Court, senate, or congressional contexts had an even lower proportion of gestures (*n* = 63, 7%), followed by semi‐scripted interviews or conversations, including teleshopping programming (*n* = 33, 4%), and finally, scripted contexts such as adverts, sitcoms, or music videos (*n* = 19, prop = 2%).

### 4.2 Statistical models

**Fig. 7:**
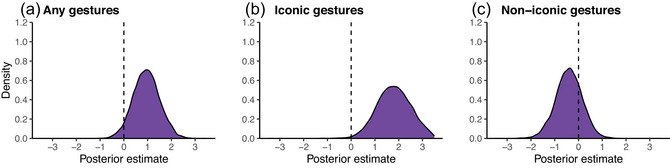
Posterior distributions for the coefficients of the word type effect (high in iconicity vs. low in iconicity), controlling for word frequency, in separate models for (a) any gestures, (b) iconic gestures only, and (c) noniconic gestures only.

The mixed logistic regression model with random intercept for word and video and a fixed effect of “type” (high in iconicity vs. low in iconicity) and word frequency revealed a positive effect of high iconicity words on the presence of gestures (logit coefficient = +0.94, *SE* = 0.56), whose 95% credible interval barely included zero [−0.11, +2.08]. The corresponding posterior distribution, indicating the range of plausible coefficient values, is shown in Fig. 7a. The posterior probability of the coefficient being of the same sign was 0.96.

The same model fitted on iconic gestures only revealed an effect of word type as well, albeit one stronger in size than that of the model on all gestures (logit coefficient = +1.89, *SE* = 0.76), with the 95% credible interval that firmly excluded zero [+0.46, +3.47], as shown in Fig. 7b. The posterior probability of the coefficient being of the same sign was 1.0. In contrast to this, the effect of word type was inconclusive when the same model was fitted on the count of all noniconic gestures. Here, the sign of the coefficient was negative (logit = −0.40, *SE* = 0.54), indicating that there was a tendency for the low iconicity words to have a slightly *higher* proportion of noniconic gestures. However, the 95% credible interval of this coefficient firmly overlapped with zero, [−1.47, +0.67], and the posterior probability of the effect being of the same sign (negative) was 0.77 (see Fig. 7c for posterior distribution).

In all three models, there was little indication that word frequency played any major role in determining gesture frequencies. For the model fitted on all gestures, the word frequency coefficient was close to zero (logit estimate = +0.03, *SE* = 0.25), including a 95% interval that firmly covered zero in a near‐symmetric fashion [−0.46, +0.52]. The posterior probability of this coefficient being the same sign was 0.55. For the model fitted on iconic gestures only, more frequent words were associated with *more* iconic gestures (logit estimate = +0.32, *SE* = 0.34), but the 95% interval firmly covered zero [−0.32, +1.01]. The posterior probability of this coefficient being the same sign was 0.84. Finally, the model fitted on noniconic gestures similarly showed no strong frequency effect (+0.06, *SE* = 0.24), with a 95% interval near‐symmetrically focused on zero, [−0.42, +0.53], and a posterior probability of being the same sign that was close to chance, 0.60. Thus, across all three sets of data, there was no strong indication that there was an effect of word frequency.

### 4.3 Part‐of‐speech: Verbs versus adjectives

Our filtered data set contained 15 adjectives and 30 verbs, based on the dominant part of speech as determined by the SUBTLEX frequencies (Brysbaert et al., 2012). Table 3 shows the gesture proportions of the search terms in this study, separated by iconicity type and part‐of‐speech.

**Table 3:** Proportion of gestures out of the total number of eligible tokens by word‐level iconicity and part‐of‐speech as determined by the SUBTLEX frequencies (Brysbaert et al., 2012)

POS (SUBTLEX)	Word‐level iconicity	No. of words	All gestures	Iconic gestures	Noniconic gestures
**Adjective**	**Low iconicity**	7	0.490	0.125	0.365
	**High iconicity**	8	0.551	0.114	0.437
**Verb**	**Low iconicity**	16	0.583	0.102	0.480
	**High iconicity**	14	0.768	0.310	0.458

For adjectives, there was relatively little difference between the overall proportion of gestures for high iconicity words (55%) and low iconicity words (49%). For verbs, this difference was more pronounced, with high iconicity verbs having a much higher proportion of gesture (77%) than low iconicity verbs (58%). We amended our main logistic regression model with the word‐level iconicity predictor the binary fixed effects POS, and the interaction between iconicity and POS (all predictors sum‐coded to aid the interpretation of main effects in the presence of interactions). For all gestures, this analysis showed a clear numerical trend for iconicity (log odd estimate: +0.43, *SE* = 0.31, 95% CrI: [−0.16, +1.06]), with a relatively high posterior probability of being of the same sign (*p =* .94). The verb predictor also had a positive effect on overall gesture proportion (+0.44, *SE* = 0.27, 95% CrI: [−0.09, +0.98]), with a relatively high posterior probability of being of the same sign (*p =* .95). There was a weak indication of an interaction (+0.27, *SE* = 0.28, 95% CrI: [−0.26, +0.82]), with a high but relatively inconclusive posterior probability of being of the same sign (*p =* .82).

For iconic gestures specifically, high iconicity adjectives actually had a slightly lower proportion of gestures (11%) than low iconicity adjectives (13%). For verbs, in contrast, there was a pronounced difference in the anticipated direction, with high iconicity verbs occurring about three times as frequently with iconic gestures (31%) than low iconicity verbs (10%). We again amended our main logistic regression model for iconic gestures with PO and the interaction between POS and word‐level iconicity (all predictors sum‐coded). Again, iconic words showed an increased proportion of iconic gestures (+0.80, *SE* = 0.41, 95% CrI: [+0.03, +1.64]), an effect with high probability of being of the same sign (posterior probability, *p =* .98). Iconic words also showed an increase in the main effect of part‐of‐speech (+0.63, *SE* = 0.34, 95% CrI: [−0.03, +1.31]). For this model, there was a slightly stronger interaction effect, with the effect of word‐level iconicity being much more pronounced for verbs (+0.52, *SE* = 0.34, 95% CrI: [−0.15, +1.20], *p =* .94).

**Fig. 8:**
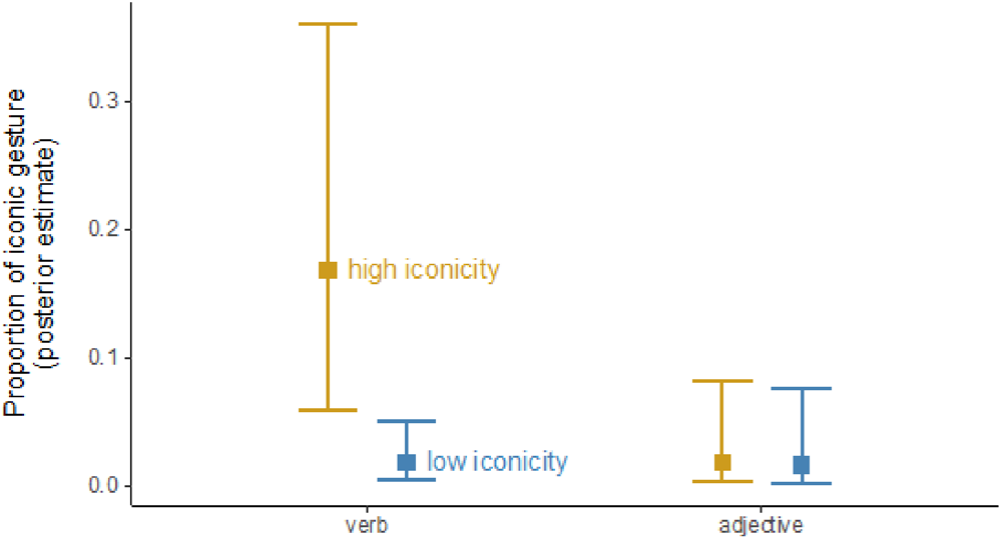
High iconicity verbs show a much higher proportion of iconic gestures than low iconicity verbs, with little difference for adjectives. Squares represent posterior means, and error bars are 95% credible intervals taken from our main mixed logistic regression model amended with the part‐of‐speech interaction.

For noniconic gestures, high iconicity adjectives had a somewhat higher proportion of gestures (44%) than low iconicity adjectives (37%), whereas high iconicity verbs (46%) had a slightly lower proportion of noniconic gestures than low iconicity verbs (48%). When we amended the model for noniconic gestures with the fixed effect of POS and the interaction, there was little evidence for any effects, with the posterior distributions of all coefficients strongly overlapping with zero (iconicity: −0.21, *SE* = 0.31, 95% CrI: [−0.85, +0.40], *p =* .75; part‐of‐speech: −0.27, *SE* = 0.28, 95% CrI: [−0.83, +0.28], *p =* .84; interaction: −0.13, *SE* = 0.28, 95% CrI: [−0.68, +0.41], *p =* .68). Fig. 8 shows the proportion of iconic gesture (posterior estimate) for verbs and adjectives by iconicity word type.

### 4.4 Sensorimotor semantics

We assessed the effect of maximum perceptual strength and maximum motor strength on gesture rates by adding these predictors as fixed effects to our original models, including the iconicity and frequency predictors. For all gestures, there was no consistent evidence for an effect of maximum perceptual strength (−0.52, *SE* = 0.44, 95% CrI: [−1.38, +0.35], posterior probability of same sign *p =* .88), or maximum motor strength (+0.02, *SE* = 0.30, 95% CrI: [−0.61, +0.57], *p* = .53), and the posterior distributions for both coefficients firmly included zero. Importantly, the iconicity effect was just as strong when these additional semantic factors were controlled for (+1.32, *SE* = 0.67, 95% CrI: [+0.03, +2.68], *p* = .98). For iconic gestures, the pattern of results looked similar, with no compelling evidence for effects of maximum perceptual strength (−0.05, *SE* = 0.57, 95% CrI: [−1.18, +1.08], *p* = .54) or maximum motor strength (−0.03, *SE* = 0.37, 95% CrI: [−0.76, +0.70], *p* = .54), but again, there was a strong effect of word‐level iconicity (+1.97, *SE* = 0.88, 95% CrI: [+0.27, +3.75], *p* = .99). For noniconic gestures, maximum perceptual strength (+0.51, *SE* = 0.43, 95% CrI: [−0.32, +1.35], *p* = .89) and maximum motor strength (+0.03, *SE* = 0.28, 95% CrI: [−0.54, +0.58], *p* = .54) also did not show compelling evidence for an effect, and similar to our main analysis, for this type of gesture, there was also no strong effect of word‐level iconicity (−0.79, *SE* = 0.64, 95% CrI: [−2.11, +0.44], *p* = .90). It is interesting to observe, however, that for these noniconic gestures only, there is a clearer trend for words with higher maximum perceptual strength to have slightly higher gesture proportions; this trend was absent for iconic gestures.

## 5 Morphosyntactic integration

Although, overall, we found that high iconicity search terms had higher rates of iconic gesture, it was not the case that all high iconicity search terms in our study had high iconic gesture rates, and that all low iconicity search terms had low iconic gesture rates. To further investigate the relationship between iconicity rating and iconic gesture rate, we re‐examined tokens from some of the high iconicity words, transcribing the co‐occurring speech and annotating the morphosyntactic behavior of the search term, including the degree of morphosyntactic integration of the terms.

### 5.1 Methods

To analyze the role of morphosyntactic integration, we focused on the three high iconicity words which had the highest iconic gesture rates (*bang* at 83%, *squish* at 81%, and *wobbly* at 60%), the three which had the lowest iconic gesture rates (*barking*, *plump*, and *saggy*, all at 0%), and three which had moderate iconic gesture rates (*woof* at 38%, *chomp* at 36%, and *zap* at 33%). For each search term, we transcribed all tokens where a gesture could have occurred (i.e., where the speaker was on screen and their hands were visible and free to gesture) and coded the utterances according to five variables of the morphosyntactic behavior of the search term within the utterance. These were: part‐of‐speech, morphological inflection/derivation, occurrence with a quotative verb, position within the utterance, and immediate repetition of the term. Table 4 shows four examples of utterances and how they were coded for each variable.

**Table 4:** Example utterances (with search term in bold) and how they were coded for each morphosyntactic coding category

Example utterance	Part‐of‐speech	Morphology	Quotative verb	Position in clause	Immediate repetition
***chomping*** *into other players*	Verb	Yes	No	Left edge	No
*they booted a passenger over his* ***saggy*** *pants*	Adjective	Yes	No	Neither edge	No
*you saw protests called* ***zaps***	Noun	Yes	No	Right edge	No
*I hear it go* ***zap zap zap zap zap***	Interjection	No	Yes	Isolation	Yes

The four categories for part‐of‐speech were: noun, adjective, verb, and interjection. We coded the search term based on how it functioned in the utterance, rather than how it was categorized according to the SUBTLEX frequencies. We used morphology and position within the utterance to inform decisions for difficult‐to‐code tokens.

For the morphology category, we asked whether the word had any inflectional or derivational suffixes. While some search terms, such as *chomp*, did not already have inflectional or derivational suffixes, some search terms, such as *saggy*, did. Hence, the decision was made based on the root of the search term rather than the exact search term.

For the quotative verb category, we coded whether the search term was introduced by a quotative verb, such as *say*, *like*, or *go*. Sometimes the search term was used in a quotation, but there was no quotative verb present, known as a “zero quotative” (Blackwell et al., 2015). For example, one speaker, while discussing their co‐presenter barking like a dog, said “*woof* does not count.” Here, the search term *woof* is a quotation, but there is no quotative verb used. Examples such as this were coded as having no quotative verb.

We also categorized the utterances based on where the search term appeared within a clause: in the leftmost position, in the rightmost position, in neither position (i.e., embedded within the utterance), or in total isolation.

Finally, we coded the utterances for whether the search term was immediately repeated. If the search term was repeated but not in succession, such as the word *squish* in the utterance “those valves have to close, squish, close, squish, close,” then this was not coded as immediate repetition. There was one instance of ablaut reduplication, in the utterance “we have to talk about Rory McIlroy, wibbly‐wobbly, kind of, to say the least,” which we coded as immediate repetition.

### 5.2 Results

Table 5 shows the percentage of tokens for each of the search terms that fit into each of the categories.

**Table 5:** Percentage of tokens that fit into the morphosyntactic categories for each word type: high iconicity words with low iconic gesture rates, high iconic gesture rates, and moderate iconic gesture rates

		Low gesture words	High gesture words	Moderate gesture words
**POS**	**Noun**	12%	0%	8%
	**Adj**.	50%	40%	8%
	**Verb**	38%	33%	51%
	**Int**.	0%	27%	34%
**Morphology**	**Yes**	75%	70%	43%
	**No**	25%	30%	57%
**Quotative verb**	**Yes**	0%	7%	16%
	**No**	100%	93%	84%
**Position in clause**	**Embedded**	77%	45%	48%
	**Left edge**	11%	25%	5%
	**Right edge**	12%	10%	16%
	**Isolated**	0%	20%	31%
**Repeated**	**Yes**	0%	24%	28%
	**No**	100%	76%	72%

Looking at POS, the search terms which had low iconic gesture rates (*saggy*, *plump*, and *barking*) were most often used as adjectives (50%), followed by verbs (38%), and finally nouns (12%). Low gesture words were never used as interjections. Similarly, search terms which had high iconic gesture rates (*bang*, *squish*, and *wobbly*) were most often used as adjectives (40%), followed by verbs (33%), and finally interjections (27%). These words were never used as nouns. Search terms which had moderate iconic gesture rates (*woof*, *chomp*, and *zap*) were most often used as verbs (51%), followed by interjections (34%), and finally nouns and adjectives (8%, respectively).

In terms of morphology, low gesture search terms more often appeared with inflection or derivation than without (75% vs. 25%). High gesture words also appeared more often with inflection or derivation, though the difference is marginally less pronounced (70% vs. 30%). Moderate gesture words appeared less often with inflection or derivation (43% vs. 57%).

Although all word types occurred more often without a quotative verb than with, there were still differences to be seen. Low gesture words were never introduced by quotative verbs. High gesture words occurred with quotative verbs more often than low gesture words (in 7% of tokens), while moderate gesture words occurred with quotative verbs most often of the three word types (in 16% of tokens).

Focusing on the search term's position within a clause, low gesture search terms were most often embedded, that is, were neither at the left nor right edge of a clause (77%). Comparatively, they were infrequently used at the right edge (12%) or at the left edge (11%) of the clause. These words never appeared in total isolation. High gesture words were also most often embedded in a clause (45%), though this was less common than in low gesture words. Like low gesture words, high gesture words also infrequently occurred at the right edge (10%) or left edge (25%). However, unlike low gesture words, high gesture words were sometimes used in total isolation (in 20% of tokens). Moderate gesture words were still most often embedded in a clause (48%), but less often than low gesture words. This was followed by those used in isolation (31%), then at the right edge (16%), and finally at the left edge (5%).

Finally, low gesture search terms were never repeated. In comparison, high gesture words were repeated in 24% of tokens, and moderate gesture words were repeated in 28% of tokens.

Further to this, we were interested especially in how the gesture rates interacted with the syntactic behavior of the search terms on a token‐by‐token level. Specifically, we wanted to know whether the high gesture and moderate gesture search terms co‐occurred with iconic gestures more often when they behaved in ways which made them more morphosyntactically isolated than integrated within the utterance (i.e., they were used as interjections, they were not used with inflectional or derivational morphemes, they were introduced by quotatives, they appeared in isolation or at the edge of utterances, and/or they were immediately repeated). Table 6 shows the percentages of tokens that occurred with an iconic gesture for these search terms, depending on how they were categorized.

**Table 6:** Iconic gesture rate for high iconicity words with high iconic gesture rates and high iconicity words with moderate iconic gesture rates, separated by morphosyntactic categories

		High gesture words	Moderate gesture words
**POS**	**Noun**	N/A	0%
	**Adj**.	68%	0%
	**Verb**	85%	16%
	**Int**.	89%	67%
**Morphology**	**Yes**	80%	27%
	**No**	73%	34%
**Quotative verb**	**Yes**	50%	50%
	**No**	80%	26%
**Position in clause**	**Embedded**	57%	17%
	**Left edge**	83%	0%
	**Right edge**	83%	0%
	**Isolated**	83%	69%
**Immediate repetition**	**Yes**	89%	67%
	**No**	76%	19%

*Note*. N/As indicate that there were no words in that category. Low iconicity words were not included because they all had an iconic gesture rate of 0%.

For both high gesture and moderate gesture search terms, iconic gesture rates were highest when they were used as interjections (89% and 67%, respectively). For high gesture words, the iconic gesture rate was similar for verbs (85%), and lowest for adjectives (68%). Moderate gesture words had iconic gesture rates of 16% when used as verbs, and 0% when used as nouns or adjectives.

When looking at the morphology of the search term, the iconic gesture rate of high gesture tokens was slightly higher when they were used with inflectional or derivational morphemes (80% vs. 73%). Among moderate gesture search terms, tokens without inflection or derivation had higher iconic gesture rates than those with (34% vs. 27%).

Somewhat surprisingly, for high gesture tokens, those which were not introduced by a quotative verb had a higher iconic gesture rate than those which were (80% vs. 50%). For moderate gesture words, the results were quite different: tokens with a quotative verb occurred with iconic gestures almost twice as often as those without (50% vs. 26%).

Looking at the position of the search terms within the clause, iconic gesture rates were the same for high gesture tokens, which were used in isolation, at the left edge only, or at the right edge only (all 83%), while those embedded in the clause had a lower iconic gesture rate (57%). Moderate gesture tokens, which were used in isolation, had the highest rate of iconic gesture (69%), followed by those embedded in a clause (17%). Those that were used at either the left edge or right edge of a clause never co‐occurred with an iconic gesture.

Finally, high gesture words had a slightly higher rate of iconic gesture when they were immediately repeated (89% vs. 76%). The same was true for moderate gesture words, but the difference is more pronounced (67% vs. 19%).

## 6 Discussion

Multimodal approaches to language take gesture to be an integral component of spoken communication, but have often emphasized a semiotic division of labor in which words are treated as arbitrary—communicating the propositional content of a message—and co‐speech gestures as iconic—communicating the imagistic and analog aspects of a message (e.g., Holler & Levinson, 2019; McNeill, 2005). However, growing recognition that many spoken words are iconic has blurred the line between arbitrary words and iconic gestures. In this study, we investigated whether English speakers produce more iconic gestures when using iconic words, similar to the use of ideophones in other languages. To determine the specific role of iconicity, we further examined whether the words’ sensory‐ and motor‐related semantics affected the frequency of co‐speech iconic gestures. Finally, we explored whether the grammatical category and syntactic positioning of iconic words affected speakers’ propensity to produce accompanying iconic gestures.

Using data from the TV News Archive, we conducted a large‐scale quantitative study comparing how often English speakers produced iconic gestures when using words rated as high or low in iconicity. After filtering, the search terms included in our data consisted of 45 verbs and adjectives: 22 high in iconicity, such as *swoosh*, *crispy*, and *slushy*, and 23 low in iconicity, such as *tamper*, *discern*, and *absent*. These were balanced, as much as possible, for perceptual strength, part‐of‐speech, and syllable length. The filtered dataset consisted of 1546 tokens, with 882 gestures, including 212 iconic gestures.

Overall, the results showed that high iconicity words co‐occurred with more gestures than low iconicity words, and specifically that high iconicity words co‐occurred with more *iconic* gestures than low iconicity words. There was little difference between the proportion of other (i.e., noniconic) gestures produced alongside high iconicity and low iconicity words. These patterns were confirmed by mixed logistic regression models that controlled for word and video. In these models, the words’ frequencies had no effect on gesture rates, regardless of whether these were overall gesture rates, iconic gesture rates, or noniconic gesture rates. The models also showed that iconic words were associated with iconic gestures, even when controlling for the words’ action strength and perceptual strength, showing that a word's iconicity was a better predictor of whether it will co‐occur with an iconic gesture than its sensorimotor‐related semantics. However, when incorporating part‐of‐speech into the analysis, we found that the effect of word‐level iconicity on gesture production was stronger for verbs than for adjectives, suggesting that the dynamic nature of verbs increases the likelihood of co‐occurring iconic gestures. When zooming in on the morphosyntactic behavior of high iconicity words in context, we found that iconic words were more likely to occur with iconic gestures when they were syntactically isolated from other words in the utterances, such as in the use of quotation. Altogether, these results show a strong association between the use of iconic words and the production of co‐speech iconic gestures.

While some have asserted that English lacks ideophones (Diffloth, 1972; Kita, 1997; Liberman, 1975; Nuckolls, 2004, 2004), research into iconic English words has found that they share many similarities to ideophones in other languages (Winter et al., 2023). Like ideophones (Akita, 2011; Dingemanse, 2019; Ibarretxe‐Antuñano, 2006; Nuckolls, 1995), iconic English words express sensory meanings (Lupyan & Winter, 2018; Sidhu & Pexman, 2018; Winter et al., 2023, 2017). Like ideophones (Akita, 2011; Childs, 1995; Dingemanse, 2019; Ibarretxe‐Antuñano, 2017; Nuckolls, 2020), iconic English words tend to be structurally marked, for example, by unusual phonotactics (Dingemanse & Thompson, 2020; Winter et al., 2023). Like ideophones, which are often associated with playful and informal discourse (Kim, Winter, & Brown, 2021; Klamer, 2002; Samarin, 1970), iconic English words often have playful and humorous connotations (Dingemanse & Thompson, 2020; Winter et al., 2023). And like ideophones (Ibarretxe‐Antuñano, 2017; Yoshida, 2012), iconic English words tend to be among the words learned earliest by children (Perry et al., 2015, 2018). Our findings from the current study highlight another similarity between iconic English words and ideophones: both occur frequently with iconic gesture.

**Fig. 9:**
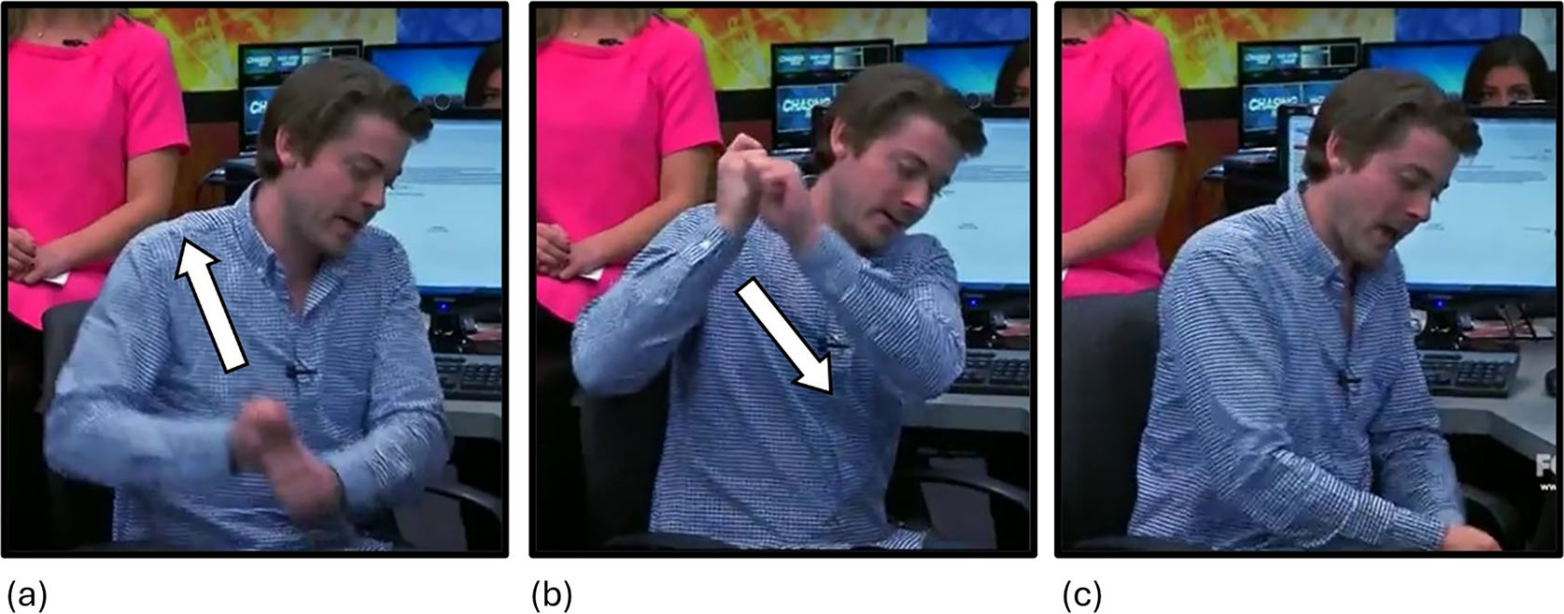
Example of an iconic enactment gesture, where the speaker mimics using a sledgehammer to make a banging noise. (a) Pre‐stroke and preparing the gesture, the speaker's hands are formed in two vertical fists, right hand above the left hand, as though holding a two‐handed tool. They begin at hip level, and he raises them. (b) “***bang‐***” The speaker's fists are at head height, now side‐by‐side, and, performing the stroke of the gesture, he brings them back down vertically. (c) “***‐ing*** away.” The speaker's hands stop at hip level. Bold italics indicate speech that co‐occurs with the stroke phase of the respective gesture. The superimposed arrow indicates the path of movement.

Although the analyses we conducted here focused on iconicity at the word level, words, of course, do not carry an iconicity rating in a person's mental lexicon that causes them to produce more gestures when using high iconicity words. Research on the integration of speech and gesture suggests that gestures are associated with phrases, or “conceptual affiliates” (De Ruiter, 2000, p. 291), driven by meaning that does not correspond directly with individual words. Despite this, our study shows that word‐level iconicity is a robust predictor of when speakers produce iconic gestures. We can interpret their co‐occurrence with iconic words not as a case of word‐level iconicity triggering gesture‐level iconicity, but as evidence that iconicity is multimodal, rather than modality‐specific. Taking this approach, iconic gestures could co‐occur with iconicity at the word level as both the iconic word and iconic gesture are produced as integrated parts of a more holistic multimodal depiction. An example of such a multimodal depiction can be seen in Fig. 9, which shows a speaker enacting a sound‐related movement, using an iconic gesture alongside the high iconicity word *bang*. The speaker enacted the action that created the banging sound, by miming holding a two‐handed tool, such as a sledgehammer, bringing it above his head and back down, as though hitting something with it. The term *bang* co‐occurred with the main stroke of the gesture. Thus, the speaker appears to have entered a multimodal depictive mode whereby he depicted both the sound of the action, using the iconic word *bang*, and the sight of the action, using the iconic gesture.

One interpretation of our results is that the observed co‐occurrence of iconic gestures with iconic words is evidence that the iconicity of the word is psychologically active during the utterance. We can draw parallels here with research on simultaneous metaphors in spoken words and gestures. Müller (2008) argued that when speakers exhibit the same conceptual metaphor in both speech and gesture, this reflects that the underlying metaphor is psychologically active during word use, rather than being a fossilized remnant of past metaphorical usage. Similarly, the use of spontaneous iconic gestures alongside iconic words could suggest that the iconicity of words is psychologically activated, despite these words functioning simultaneously as conventionalized forms. On this view, the form‐meaning resemblance of words may not always be salient to the speaker, but can become activated in the context of a particular utterance. Further evidence that the iconicity of a word is active in an utterance may be seen when they are produced with iconic modulations that enhance their iconicity, such as through iconic prosody (Perlman, in press). For example, English speakers have been shown to modulate the duration, fundamental frequency, and voice quality of words to mimic their meaning in context (Perlman, Clark, & Johansson Falck, 2015; Shintel, Nusbaum, & Okrent, 2006).

Theories such as the GSA framework posit that gestures emerge as part of the perceptual and motor imagery that is activated during thinking‐for‐speaking. Such theories predict that a word's perceptual or action strength rating would be an important factor in the likelihood that the use of a word co‐occurs with the production of an iconic gesture. This is supported by research which has found that people produce gestures most frequently when communicating about visual or motor imagery than abstract concepts (Feyereisen & Havard, 1999; Hostetter & Alibali, 2007). At odds with this theory, our results showed evidence for a tight connection between iconic words and iconic gestures, irrespective of words’ perceptual or motor strength. This supports the idea that the high iconic gesture rates for high iconicity words is due to their iconic nature, rather than due to iconic words being more prone to expressing perceptual semantics (e.g., Lupyan & Winter, 2018; Sidhu & Pexman, 2018; Winter et al., 2023, 2017). On this view, speakers produce iconic gestures when they are in a depictive mode that tends to be engaged by iconic words, and are influenced less by the particular meaning that is being expressed.

However, while intriguing, these results should be taken with some caution. It is important to recognize the limitations of the sensorimotor ratings, which—like iconicity ratings—are subjective, rather than objective, measures that were provided by participants divorced from communicative context. For example, the words *munch* and *ordain* both have maximum perceptual strength ratings of 2.7, though intuitively *munch* appears to be more associated with the senses than *ordain*. Similarly, *swish* and *acquaint* have maximum action strength ratings of 1.9, though *swish* appears to be more grounded in action than *acquaint*. The ratings task may lead participants’ judgments to diverge from these intuitions for (at least) two reasons. First, as the maximum strength ratings reflect the highest average score for either perceptual domain or action effector, they do not capture the richness of the sensorimotor experience that the words evoke. For example, the word *munch* might elicit a multisensory experience encompassing sound, taste, touch, and mouth action, which the participant is required to disentangle to give individual ratings. Each rating may not be high, but they combine to create a stronger sensory experience; indeed, this is reflected in the fact that *munch* has a lower modality exclusivity rating than *ordain*.

Second, in making their ratings, participants may be considering associations with the word, rather than its direct meaning. In the case of *ordain*, although its meaning may appear abstract, participants may associate it with an ordination ceremony, which is a sensorimotor experience in that the physical ritual is seen and the accompanying spoken words are heard. This is reflected in the fact that *ordain* has low perceptual ratings generally, but higher ratings for sound and vision. In the case of *acquaint*, it has low action ratings for foot/leg, but higher ratings for hand/arm, mouth, and torso, suggesting that participants may be associating the word with actions such as shaking hands, speaking, and hugging. It has a similar rating for head, which could reflect that participants associate abstract concepts with thinking, and thinking with the head. This highlights the broader difficulty of operationalizing the lexical semantics of perception and action for large‐scale quantitative studies such as this one. Summary metrics like maximum perceptual or strength rating are practical for large‐scale analyses but may overlook meaningful qualitative differences in the way words engage sensorimotor systems.

**Fig. 10:**
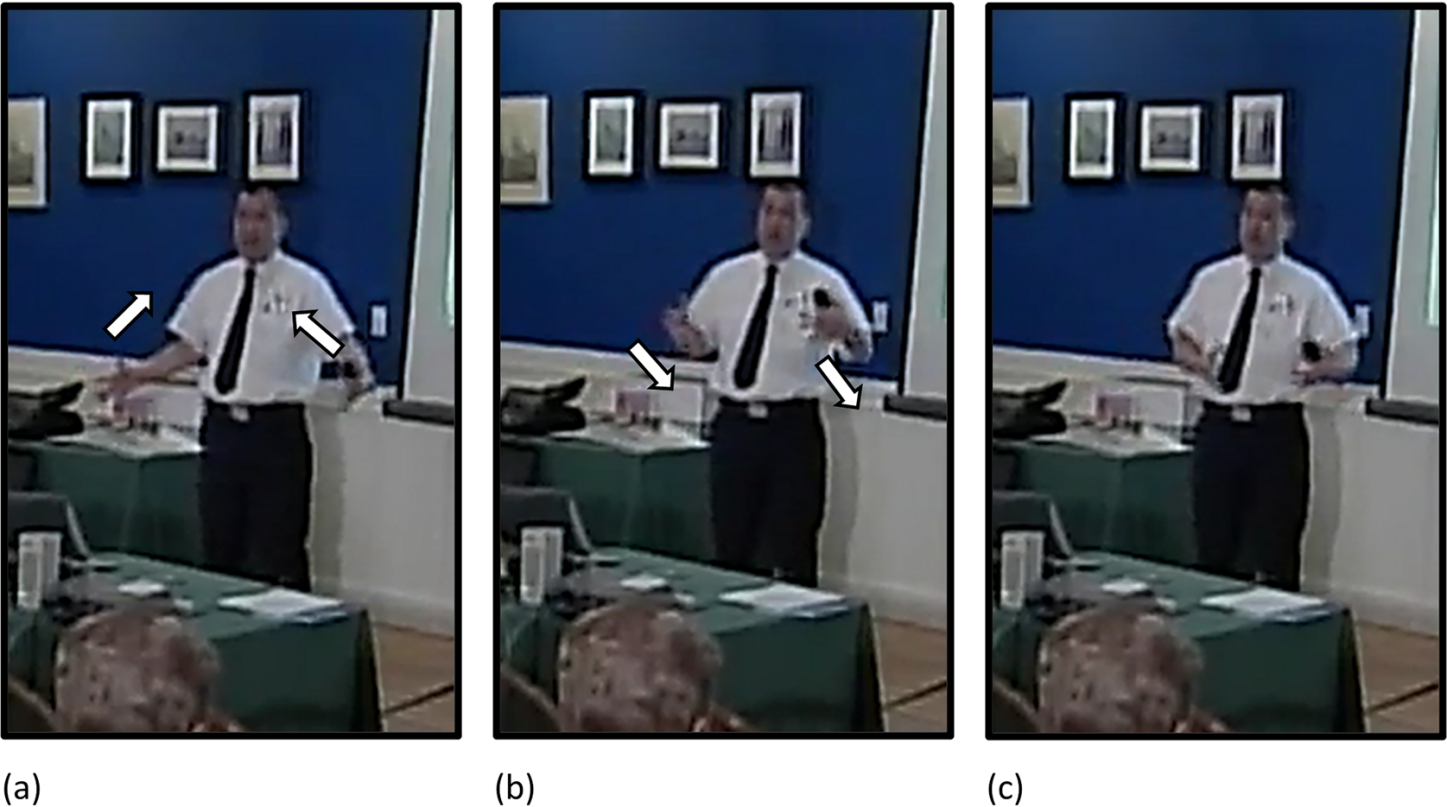
Example of an iconic depiction gesture, where the speaker traces the path of movement of an object. (a) Pre‐stroke, the speaker's open hands are held out in front of him, at about hip level. (b) “wherever you…” Pre‐stroke, the speaker prepares the gesture by raising both hands and bringing them inward, still in an open shape, to chest level. (c) “… ***put it***” In the stroke of the gesture, the speaker moves both hands, retaining the open hand shape, left and downward, stopping at stomach level. Bold italics indicate speech that co‐occurs with the stroke phase of the respective gesture. The superimposed arrow indicates the path of movement.

While our analysis did not show an effect of sensorimotor semantics, our results did reveal that high iconicity verbs in particular were far more likely to co‐occur with iconic gestures than low iconicity verbs, whereas high iconicity adjectives had a slightly lower proportion of iconic gestures than low iconicity adjectives. An amended logistic regression model, which accounted for part‐of‐speech, showed that the effect of iconicity was much more pronounced for verbs than adjectives. Furthermore, despite our overall finding that iconic words were associated with iconic gestures, there were low iconicity search terms in our study, which also had high iconic gesture rates—particularly the verb *put*. Most iconic gestures that occurred alongside the verb *put* were depictions where the speaker traced the path of movement of an object. For example, in Fig. 10, the speaker depicts a sideways downward movement while saying “wherever you put it,” iconically representing the action of moving something and putting it down.

We theorize that the effect of iconicity may be stronger for verbs than for adjectives, and that some low iconicity verbs had high iconic gesture rates, because verbs represent more dynamic concepts than adjectives, which tend to have more static meanings (Givón, 1984; Langacker, 2008; Strik Lievers & Winter, 2018). Hence, verbs’ association with action and movement may make them more prone to co‐occurring with iconic gestures than adjectives, including low‐iconicity verbs like *put* that are strongly associated with movement. The relationship between action concepts and gesture is supported by evidence from ideophone research; Dingemanse (2013) found that ideophones which relate to movement occur more often alongside iconic gestures than ideophones which relate to, for example, temperature or inner feelings. He argued that this could have contributed to the very high gesture rate in studies of Japanese ideophones (Kita, 1993, 1997) which referred mainly to the domains of space and movement, and thus, may have been more easily expressed in gestures (Dingemanse, 2013). Our finding that verbs—as words that tend to be related to action—are more prone to gesture is also congruent with the GSA framework and the theory that iconic gestures are a result of the sensorimotor simulations formulated as part of thinking‐for‐speaking (Hostetter & Alibali, 2008, 2019).

Despite our main finding that high iconicity words occur more often with iconic gestures than low iconicity words, there were some high iconicity words that co‐occurred with few or no iconic gestures. This variation prompted us to investigate what additional factors might influence the production of iconic gestures. Previous research has found that ideophones are more likely to co‐occur with iconic gestures in syntactic contexts that highlight and enable multimodal depictive performances, that is, when they are more syntactically isolated within an utterance (Dingemanse & Akita, 2017). Relatedly, the production of iconic gestures is common in the use of quotation constructions, where quotative verbs serve to mark and isolate the multimodal depiction of speech acts and other events (Clark & Gerrig, 1990). We, therefore, explored whether the morphosyntactic behavior of the high iconicity words in our study influenced their propensity to co‐occur with iconic gestures by analyzing a subset of high iconicity words with high, moderate, and low iconic gesture rates. Consistent with Dingemanse and Akita's (2017) findings, markers of syntactic isolation were more frequent in iconic words with high and moderate gesture rates compared to those with low gesture rates. Words with high and moderate gesture rates were used more often as interjections, were less often modified with inflectional and derivational morphemes, were more often used in quotations, were more frequently isolated within a clause, and were more often repeated. Intriguingly, words with moderate iconic gesture rates exhibited these markers of syntactic isolation more frequently than those with high gesture rates, and these syntactic markers appeared to be more tightly connected with iconic gesture production for moderate gesture words than for high gesture words. This suggests that when iconic words have a weaker inherent association with gestural expression, speakers may be more inclined to mark the activation of iconicity by employing quotation and other features of syntactic isolation, which also serve to create the pragmatic and prosodic space necessary to facilitate multimodal depiction.

Finally, we consider the overall gesture rate in our study across all words—both adjectives and verbs—which was notably high at 62%. Other studies using data from TV news broadcasts have also found high gesture rates. Pagán Cánovas et al. (2020) found that people gestured 69% of the time alongside temporal expressions, and Alcaraz‐Carrión et al. (2022) found that people gestured 61% of the time when using phrases related to adding and subtracting, both using the Red Hen Lab NewsScape Library. Similarly, Woodin et al. (2020), using the TV News Archive, reported a 78% gesture rate for phrases expressing numerical magnitude. However, when interpreting the gesture rates from our study in relation to those from other gesture studies, it should be noted that the use of different data filtering processes or different standards for “eligible” tokens has the potential to change the gesture rate. For example, our study included tokens where the speaker's hands were not directly visible, but it was nonetheless evident that they were not gesturing, such as if a speaker had their hands on a podium but their wrists were still visible. Only including tokens where the speaker's hands were in no way obscured could reduce the overall number of eligible tokens and increase the gesture rate. That caveat notwithstanding, the overall high gesture rate in our study, and other studies using TV news data, could be attributed to the narrative context of many of the videos. Research regarding gestures and ideophones has found a higher gesture rate in narrative contexts, where depiction and illustration are more common, than across other contexts (Dingemanse, 2013), and in many tokens in our data, speakers were telling stories in the form of interviews, news reports, and speeches. Gesture rate may also be affected by the televised context of the videos, in that some speakers are actors or presenters. However, coding the videos for context revealed that very few tokens where a gesture occurred were from partly or wholly scripted contexts. This suggests that a significant proportion of gestures in our data can be deemed relatively “natural” or spontaneous.

## 7 Conclusion

Taken together, our findings provide compelling evidence that iconicity operates across the spoken and gestural modalities, even in English—a language often characterized as a lower‐bound case for iconic vocabulary. When speakers use words rated high in iconicity, such as *swoosh*, *squish*, and *bang*, they produce iconic gestures at more than twice the rate observed with low iconicity words. This association persists even when controlling for potentially mediating factors like word frequency and sensorimotor semantics. Our findings point to a theory of iconicity as an inherently multimodal phenomenon: speakers appear to engage in a depictive mode in which they produce coordinated ensembles of iconic words and gestures to create rich, embodied representations of meaning. The finding that such robust iconicity‐gesture associations emerge in English, traditionally viewed as iconically impoverished, strengthens the broader case that iconicity represents a fundamental feature of human language.

## Funding information

The authors received no specific funding for this work.

## Conflict of interest statement

The authors have no conflicts of interest to disclose.

## Ethics approval statement

The authors confirm that the ethical policies of the journal, as noted on the journal's author guidelines page, have been adhered to. No ethical approval was required as no original data was collected.

## Data Availability

The data that support the findings of this study are openly available in the Open Science Framework at https://osf.io/3myx4/.
